# Prognostic value of acute cor pulmonale in COVID-19-related pneumonia: A prospective study

**DOI:** 10.3389/fmed.2022.824994

**Published:** 2022-10-04

**Authors:** Christophe Beyls, Nicolas Martin, Thomas Booz, Christophe Viart, Solenne Boisgard, Camille Daumin, Maxime Crombet, Julien Epailly, Pierre Huette, Hervé Dupont, Osama Abou-Arab, Yazine Mahjoub

**Affiliations:** ^1^Department of Anesthesiology and Critical Care Medicine, Amiens University Hospital, Amiens, France; ^2^UR UPJV 7518 SSPC (Simplification of Care of Complex Surgical Patients) Research Unit, Jules Verne University of Picardie, Amiens, France

**Keywords:** acute cor pulmonale (ACP), COVID-19, AVDS, speckle tracking, ARDS

## Abstract

**Background:**

It is known that acute cor pulmonale (ACP) worsens the prognosis of non-coronavirus disease 2019 (COVID-19) acute respiratory distress syndrome (NC-ARDS). The ACP risk score evaluates the risk of ACP occurrence in mechanically ventilated patients with NC-ARDS. There is less data on the risk factors and prognosis of ACP induced by COVID-19-related pneumonia.

**Objective:**

The objective of this study was to evaluate the prognostic value of ACP, assessed by transthoracic echocardiography (TTE) and clinical factors associated with ACP in a cohort of patients with COVID-19-related pneumonia.

**Materials and methods:**

Between February 2020 and June 2021, patients admitted to intensive care unit (ICU) at Amiens University Hospital for COVID-19-related pneumonia were assessed by TTE within 48 h of admission. ACP was defined as a right ventricle/left ventricle area ratio of >0.6 associated with septal dyskinesia. The primary outcome was mortality at 30 days.

**Results:**

Among 146 patients included, 36% (*n* = 52/156) developed ACP of which 38% (*n* = 20/52) were non-intubated patients. The classical risk factors of ACP (found in NC-ARDS) such as PaCO_2_ >48 mmHg, driving pressure >18 mmHg, and PaO_2_/FiO_2_ < 150 mmHg were not associated with ACP (all *P*-values > 0.1). The primary outcome occurred in 32 (22%) patients. More patients died in the ACP group (*n* = 20/52 (38%) vs. *n* = 12/94 (13%), *P* = 0.001). ACP [hazards ratio (HR) = 3.35, 95%CI [1.56–7.18], *P* = 0.002] and age >65 years (HR = 2.92, 95%CI [1.50–5.66], *P* = 0.002) were independent risk factors of 30-day mortality.

**Conclusion:**

ACP was a frequent complication in ICU patients admitted for COVID-19-related pneumonia. The 30-day-mortality was 38% in these patients. In COVID-19-related pneumonia, the classical risk factors of ACP did not seem relevant. These results need confirmation in further studies.

## Introduction

Acute cor pulmonale (ACP) is a frequent, well-known complication of non-coronavirus disease 2019 (COVID-19) (NC) acute respiratory distress syndrome (ARDS), requiring protective mechanical ventilation. In NC-ARDS, the prevalence of ACP is 22% despite protective mechanical ventilation and is an independent risk factor for mortality (1). COVID-19-related ARDS seems atypical (2) and needs specific management, such as advanced immune or cell therapies (3). However, data on ACP in COVID-19 are scarce. ACP is defined, using echocardiography, as an acute right ventricular (RV) dilatation (end-diastolic RV/left ventricle ratio of >0.6) associated with septal dyskinesia (4) due to increased RV afterload that may eventually lead to RV failure. In NC-ARDS, several pathophysiological mechanisms, such as hypoxic pulmonary vasoconstriction, hypercapnia, or positive pressure ventilation, induce an elevation of pulmonary vascular resistance (PVR), leading to an increased RV afterload (5).

To assess the risk of ACP under mechanical ventilation, a clinical risk score was previously developed (1). ACP was associated with both right and left ventricular dysfunction due to ventricular interdependence: RV dysfunction leads to RV dilatation and leftward septal shift, restricting the left ventricle and decreasing cardiac output. ACP is reversible, so early diagnosis of ACP is of utmost importance for ventilator setting adaptation and clinical management to avoid hemodynamic instability related to RV dysfunction (6).

The clinical presentation of ARDS related to COVID-19 infection (CARDS) differs from NC-ARDS (7, 8). CARDS presents some “atypical” features (9), including high lung compliance, low recruitability (10), increased cardiac output with low PVR (11), and increased intrapulmonary shunting (12). In this situation of “acute vascular distress syndrome” (AVDS) (13), the increase in pulmonary blood flow and the alteration in PVR may affect RV afterload and promote RV dilation and dysfunction. Several echocardiographic studies have shown that RV dilatation (14) and RV dysfunction (15) were associated with a poor prognosis in COVID-19 infection. However, in these studies, the presence of ACP was rarely reported (14–16) except in cases of pulmonary embolism (PE) (17). Besides, some authors showed that classical factors of the ACP risk score were not associated with ACP, probably due to different pathophysiology (18). There are few data on ACP assessed by transthoracic echocardiography (TTE) in patients with COVID-19-related pneumonia requiring ICU hospitalization.

The aim of this study was to evaluate the incidence, 30-day mortality, and clinical factors associated with ACP evaluated with a TTE in a cohort of patients hospitalized in the ICU for COVID-19-related pneumonia. We hypothesized that ACP is a frequent manifestation of COVID-19-related pneumonia even in non-intubated patients (under high flow oxygen or non-invasive ventilation). Moreover, we also hypothesized that ACP increases 30-day mortality.

## Materials and methods

### Population

Between 1 March 2020 and 1 June 2021, adult patients (> 18 years of age) were admitted to the ICU at Amiens University Hospital for hypoxemic pneumonia related to SARS-CoV-2 infection, with a TTE performed within 48 h of ICU admission, and were prospectively included in the study. Exclusion criteria were patients with permanent atrial and ventricular pacing, pregnant women, patients under extracorporeal membrane oxygenation (ECMO), those with supraventricular tachycardia during the TTE exam, and those with poor image quality for RV analysis. Patients were included on the day of the TTE examination.

### Ethics

This is an ancillary study of a prospective cohort study of patients with COVID-19 infection hospitalized in the ICU at Amiens University Hospital (NCT04354558). This study was approved by the Amiens University Hospital IRB (Comité de Protection des Personnes Nord-Ouest II CHU–Place V. Pauchet, 80054 AMIENS Cedex 1, CNIL Number: PI2020_843_0026). In accordance with French law on clinical research for non-interventional studies, informed consent was waived but oral and written information was provided whenever possible to the patients and their families, specifying that they could oppose using their data (19).

### Data

Electronic data, medical reports, and biological values were collected prospectively. SARS-CoV-2 infection was confirmed by a positive reverse transcription-polymerase chain reaction on a nasopharyngeal swab or bronchoalveolar lavage on ICU admission. The severity of illness at the time of the TTE exam was evaluated by the simplified acute physiology score (SAPS) II (20). The severity of COVID-19-related pneumonia was defined according to the World Health Organization (WHO) case definition (21). The critical group included patients with respiratory failure requiring mechanical ventilation and shock or organ failure (21). Vasopressor use was evaluated by the SOFA cardiovascular (SOFA cv) score (22). The ACP risk score was defined by four variables, namely, pneumonia as cause of ARDS, driving pressure >18 cmH_2_O, PaO_2_/FiO_2_ ratio <150 mmHg, and PaCO_2_ level > 48 mmHg (1). A chest computed tomography scan assessed radiological lung involvement and PE with intravenous contrast injection before ICU admission. Outcomes during ICU stay and vital status at 30 days were collected. The primary outcome was mortality at day 30. Regarding anticoagulation, all patients received intravenous unfractionated heparin (Heparin Choay^®^, Sanofi-Aventis, France) for an anti-factor Xa activity target of 0.20–0.40 U/ml to reduce COVID-19-associated risk of thrombosis.

### Echocardiography

Trained operators performed TTE in the supine position within 48 h of ICU admission. Standard echocardiography protocol was used according to the American Society of Echocardiography guidelines (23) and the European Society of Cardiology (24). Echocardiographic images were obtained through a high-quality, commercially available ultrasound system (CX50, Philips Healthcare). All operators had a level III competence in general adult TTE (25). Caution was taken for a complete analysis of the RV with the good delimitation of the endocardium border and RV-free wall. Conventional RV systolic parameters such as tricuspid annular plane systolic excursion (TAPSE), tricuspid S wave (RV-S’), and RV fractional area change (RV-FAC) were assessed in apical four-chamber view as recommended (23). RV dilatation was defined when the end-diastolic RV/LV area ratio was >0.6 in a four-chamber or subcostal view (23). In TTE, ACP was defined as RV dilatation (end-diastolic RV/LV area ratio of >0.6) associated with septal dyskinesia (4). RV systolic function was assessed using conventional parameters (TAPSE, RV-S’, and RV-FAC) and bidimensional speckle tracking parameters such as tricuspid annular displacement (TAD). TAD parameters were composed of (1) TAD lateral, (2) TAD septal, and (3) RV longitudinal shortening fraction (RV-LSF). TAD parameters were measured in a focused RV four-chamber view and calculated with a dedicated software described in a previous study (26).

### Statistical analysis

Data are expressed as mean ± standard deviation (SD), median [interquartile range], or numbers (percentage), as appropriate. Comparisons between the two groups used the chi-square test or Fisher’s exact test for categorical variables and Student’s *t*-test or the Mann–Whitney–Wilcoxon test, as appropriate, for continuous variables.

To evaluate independent factors associated with ACP, univariate logistic regression was performed with classical factors, such as PaO_2_/FiO_2_ <150 mmHg, driving pressure >18, and PaCO_2_ > 48, which were included in the ACP risk score (1). We also included in the logistic regression variables such as body mass index (BMI), diabetes, PE, and mechanical ventilation reported to be potentially associated with ACP (18).

The multivariate Cox regression model was used to identify the parameters associated with the primary outcome and calculate the hazard ratio (HR) and 95% confidence interval (CI). Variables with a probability of <0.10 were integrated into the multivariate analysis. The Kaplan–Meier method was used to plot the survival curves compared with the log-rank test.

A statistical test was significant when the P-value was less than 0.05. All P-values are the results of 2-tailed tests. Statistical analyses were performed using the SPSS software version 24 (IBM Corp, Armonk, NY).

## Results

**FIGURE 1:**
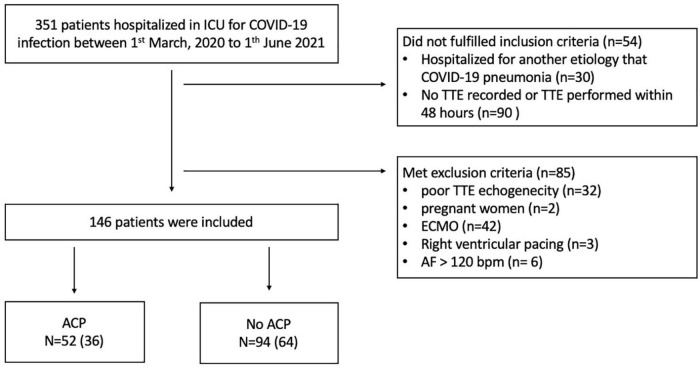
Flow diagram of the study group. ACP, acute cor pulmonale; AF, atrial fibrillation; ECMO, extracorporeal membrane oxygenation; ICU, intensive care unit; TTE, transthoracic echocardiography.

A total of 351 patients were admitted to the ICU at Amiens University Hospital for COVID-19-related pneumonia during the study period. Notably, 228 (65%) patients had a TEE within 48 h of ICU admission, and 82 (36%) had exclusion criteria (42 (51%) patients received ECMO and 32 (39%) patients had a poor TTE echogenicity). A total of 146 patients were included in the study. Patients were divided into two groups according to the presence or absence of ACP diagnosed by TTE. ACP was diagnosed in 52 out of 146 (36%) patients and was absent in 94 out of 146 (64%) patients (Figure 1).

### Clinical and demographic characteristics of patients

There was no significant difference between the two groups regarding age, clinical presentation, and biology at inclusion (Table 1). Of the 146 patients, 135 (92%) had chest CT angiography before ICU admission. Only 2 (4%) patients had a PE in the ACP group before ICU admission.

**TABLE 1:** Demographics and clinical data.

Variables	No ACP (*n* = 94)	ACP (*n* = 52)	*P* value
Age (years)	60 [58-68]	59 [58-68]	0.33
BMI (kg.m^–2^)	29.8 [25.7-34.1]	30.7 [26.3-35.7]	0.51
SAPS II score at inclusion	33 [21-57]	38 [24-58]	0.67
Male gender (*n;%*)	67 (71)	37 (71)	0.75
**Medical history** None Hypertension Diabetes Dyslipidemia Smoking (former or active) Chronic renal disease COPD/asthma Coronary or peripheral artery disease	12 (13) 51 (54) 30 (32) 27 (29) 16 (17) 8 (9) 9 (10) 1 (1)	9 (17) 23 (44) 12 (23) 17 (33) 14 (27) 5 (10) 9 (17) 4 (8)	0.46 0.3 0.34 0.71 0.2 1 0.19 0.77
**Chronic treatment** Statin Beta blocker ACE inhibitor ARBs	29 (31) 20 (21) 19 (20) 17 (18)	12 (23) 13 (25) 11 (21) 8 (15)	0.34 0.68 1 0.82
**Time to first symptom to ICU admission (days)**	8 [6-11]	7 [4-9]	0.60
**CT scan (*n* = 135/146)** Frosted glass Condensation Crazy Paving Lung involvement > 50% Pulmonary embolism	84 (89) 55 (59) 24 (26) 43 (46) 6 (6)	51 (98) 29 (56) 11 (21) 23 (44) 2 (4)	0.29 0.71 0.54 0.71 0.71
**Biological investigations at inclusion** Lymphocyte count, *mm*^–^*^3^* C reactive protein, *mg l*^–^*^1^* Serum creatinine, umol/L Troponine Tc HS, *ng ml*^–^*^1^* BNP, *pg ml*^–^*^1^* Platelet count x 10^9^ l^–1^ Fibrinogen (g l^–1^) PT (%) aPTT	700 [500-900] 146 [90-201] 69 [53-88] 19 [7-53] 61 [22-123] 230 [179-391] 6.0 [4.7-7.3] 76 [70-86] 1.1 [0.9-1.2]	700 [500-900] 145 [89-263] 80 [58-112] 21 [9-57] 59 [36-142] 256 [174-305] 5.8 [5.0-7.6] 76 [66-84] 1.1 [1.0-1.2]	0.93 0.94 0.34 0.95 0.85 0.29 0.59 0.67 1

Data are presented as median [interquartile range] and number (percentage). ACE, angiotensin-converting enzyme; aPTT, activated partial thromboplastin time; ARBs, angiotensin II receptor blockers; BMI, body mass index; BNP, brain natriuretic peptide; CT, computerized tomography; COPD, chronic obstructive pulmonary disease; PT, prothrombin time. SAPS, simplified acute physiology score; WBC, white blood cell.

### Echocardiographic findings (Table 2)

Echocardiographic parameters are presented in Table 2. In the non-ACP group, 49 (52) patients had RV dilatation. In the ACP group, RV dilatation was predominant in the middle cavity (39 [35-43] mm vs. 35 [29-42] mm; *P* = 0.03). TAD parameters were more impaired in the ACP group than in the non-ACP group, especially for RV-LSF (20.4 [15.9–23.9]% vs. 22.3 [19.8–26.3]%, *P* = 0.004). There was no significant difference between the two groups for conventional parameters of RV systolic function (TAPSE, S′ wave, and RV-FAC).

**TABLE 2:** Echocardiographic data.

Overall population (*n* = 146]	No ACP (*n* = 94)	ACP (*n* = 52)	*P* value
***LV systolic parameters*** LVEF (%) LV end diastolic volume (ml) LV end systolic volume (ml) Stroke volume index (ml/m^2^) CO (l min^–1^)	63 [53-72] 92 [62-114] 31 [20-47] 30 [24-39] 4.7 [4.0-6.2]	61 [50-71] 101 [74-124] 37 [26-57] 30 [24-36] 4.7 [3.9-6.7]	0.43 0.18 0.33 0.61 0.87
***LV diastolic function parameters*** E wave (cm s^–1^) A wave (cm s^–1^) E/A ratio Lateral E/e’ E wave deceleration time (ms)	77 [66-92] 75 [58-92] 0.98 [0.72-1.25] 8.5 [6.8-11.0] 253 [185-344]	81 [67-102] 81 [63-104] 0.87 [0.73-1.41] 8.2 [6.6-10.8] 245[192-335]	0.22 0.27 0.69 0.83 0.77
LA volume (ml) LA volume index (ml/m^2^)	32 [22-44] 16 [9-20]	38 [24-51] 17 [9-24]	0.53 0.41
***RV parameters*** RV basal dimension (mm) RV mid-cavity dimension (mm) RV longitudinal dimension (mm) RV EDA (cm^2^) RV ESA (cm^2^) RV EDA/VG EDA ratio RA volume indexed to BSA (ml/m^2^)	44 [38-49] 35 [29-42] 77 [68-86] 20.0 [16.0-24.3] 11.0 [7.9-14.2] 0.66 [0.53-0.84] 15.1 [11.0-22.6]	45 [40-52] 39 [35-43] 78 [70-84] 22.8 [18.1-26.0] 12.5 [9.2-16.8] 0.86 [0.67-1.13] 19.0 [10.0-25.1]	0.98 0.03 0.63 0.04 0.03 0.0001 0.51
RV dilatation, *n (%*)	49 (52)	52 (100)	-
***RV Systolic function parameters*** TAPSE (mm) RV- S’ (cm/s^–1^) RV FAC (%)	23.5 [19.7-27] 16.1 [13.4-19.1] 44 [36-53]	22.7 [19-26.2] 16.0 [12.5-19.4] 44 [38-51]	0.85 0.60 0.68
***TAD parameters*** TAD lateral (mm) TAD septal (mm) RV-LSF (%)	21.6 [18.8-25.9] 12.8 [9.4-14.3] 22.3 [19.8-26.3]	18.6 [15.9-23.3] 9.8 [7.85-13.7] 20.4 [15.9-23.9]	0.009 0.014 0.004

Continuous variables are expressed as median [interquartile range] and number (percentage). CO, cardiac output; BSA, body surface area. EDA, end diastolic area; ESA, end-systolic area; FAC, fractional area change; LA, left atrial; LV, left ventricle; LVEF: left ventricular ejection fraction; RA, right atrium; RV: right ventricle; RV-LSF, right ventricle longitudinal shortening fraction; TAD, tricuspid annular displacement; TAPSE, tricuspid annular plane systolic excursion.

### Hemodynamic parameters, ventilator settings, and outcomes

A total of 20 (38%) patients in the ACP group were not intubated at TTE examination (Table 3). There was no difference between the two groups in the number of variables included in the ACP risk score (all *P*-values > 0.1) and in the ACP risk score itself (2 [2–2.5] vs. 2 [2.2.5], *P* = 0.12). Patients in the ACP group had more noradrenaline than in the non-ACP group (*P* = 0.01). Cardiogenic shock (P = 0.01) and extra renal replacement therapy (P = 0.05) were higher in the ACP group than in the non-ACP group.

**TABLE 3:** Clinical characteristics and outcomes of patients having COVID-19-related pneumonia with and without acute cor pulmonale.

Variables	No ACP (*n* = 94)	ACP (*n* = 52)	*P* value
**Hemodynamic parameters at inclusion** HR, *bpm* SAP, *mmHg* DAP, *mmHg* MAP, *mmHg* SpO_2_,%	85 [76- 99] 128 [113-142] 69 [60-83] 85 [70-92] 93 [90-96	81 [72-90] 130 [117-145] 67 [59-76] 85 [70-92] 93 [91-95]	0.46 0.63 0.87 0.37 0.61
**Blood gas values at inclusion** pH PaO_2_, *mmHg* PaCO_2_, *mmHg*	7.42 [7.35-7.45] 80 [60-106] 38 [33-44]	7.42 [7.32-7.46] 77 [66-87] 39 [33-47]	0.64 0.59 0.59
**Critical group at inclusion** Vasopressors use, *n (%*) Mechanical ventilation, *n (%*) PaO_2_/FiO_2_ ratio PEEP, *cmH20* Driving pressure, *cmH20* Tidal volume, *ml per kg* Compliance, *ml/cmH20* Plateau pressure, *cmH20*	20 (21) 58 (62) 91 [70-136] 12 [12-15] 14 [12-15] 5.8 [5.2-6.1] 30 [26.4-35.2] 27 [25-29]	21 (40) 32 (62) 100 [70-128] 13 [11-14] 13 [11-15] 6.1 [5.8-6.2] 31.7 [25.2-38] 28 [24-29]	0.01 0.96 0.89 0.96 0.88 0.65 0.87 0.52
**ACP risk score for patients under MV (*n*)** Pneumonia as cause of ARDS Driving pressure > 18 cmH_2_O PaO_2_/FiO_2_ < 150 mmHg PaCO_2_ > 48 mmHg Total ACP risk score (0-4)	58 58 (100) 4 (7) 43 (74) 9 (15) 2 [2-2.5]	32 32 (100) 6 (18) 24 (80) 10 (30) 2 [2-2.5]	- 0.16 0.62 0.11 0.12
**Respiratory evolution** Pneumothorax, *n (%*) Ventilator associated pneumoniae, *n (%*) ECMO, *n (%*) Tracheostomy, *n (%*) Time under MV, days	10 (11) 50 (54) 9 (10) 8 (9) 20 [12-31]	5 (10) 30 (58) 6 (11) 5 (10) 19 [5-26]	1 0.72 0.78 1 0.43
**Thrombotic complication** Pulmonary embolism, *n (%*) Deep vein thrombosis, *n (%*)	8 (9) 6 (6)	6 (11) 3 (6)	0.77 1
Renal replacement therapy	13 (14)	14 (27)	0.05
Cardiogenic shock, *n (%*)	4 (4)	9 (17)	0.01
**Outcome** Mortality at 30-days, *n (%*) ICU Mortality, *n (%*) ICU length of stay, *days*	12 (13) 15 (16) 13 [7-32]	20 (38) 24 (46) 19 [7-27]	0.001 0.001 0.71

Data are presented as median [interquartile range] and number (percentage). ACP, acute cor pulmonale; DAP, diastolic arterial pressure; ECMO, extracorporeal membrane oxygenation; HR, heart rate; ICU, intensive care unit; MAP, mean arterial pressure; MV, mechanical ventilation; PEEP, positive end-expiratory pressure; SpO_2_, pulse saturation of oxygen.

### Factors associated with acute cor pulmonale

In our study, no predictive factors for ACP were found even in the patients in the mechanical ventilation group. In univariate analysis, factors composing the ACP risk score were not associated with ACP (Table 4).

**TABLE 4:** Factors associated with acute cor pulmonale in patients with COVID-19-related pneumonia.

Variables		

Overall population	Univariate analysis	
	
	OR (95%CI)	*P* value
BMI	1.03 [0.98-1.01]	0.21
Diabetes	0.64 [0.29-1.39]	0.26
Chronic renal disease	1.14 [0.35-3.7]	0.82
Mechanical ventilation	1.11 [0.56-2.2]	0.74
Pulmonary embolism	1.33 [0.43-4.01]	0.61
*Patients under mechanical ventilation*		
PaCo2 > 48 mmHg*	2.23 [0.80-6.17]	0.12
Driving Pressure > 18 cmH_2_0*	2.61 [0.59-11.4]	0.20
PaO_2/_FiO_2_ < 150*	1.86 [0.57-6.01]	0.29

BMI, body mass index; CI, confidence interval; OR, odds ratio. *Acute cor pulmonale risk score parameters.

### Prognosis of acute cor pulmonale

**FIGURE 2:**
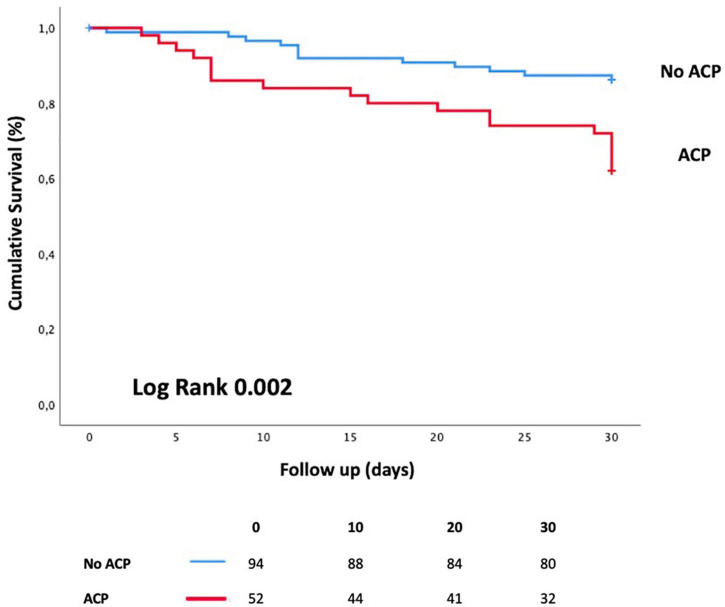
Kaplan–Meier curves showing event-free survival according to the presence of acute cor pulmonale. ACP, acute cor pulmonale.

The primary outcome occurred in 32 (22%) patients and more often in the ACP group (*n* = 20/52 vs. *n* = 12/94, *P* = 0.001). Overall, 40 (27%) patients died during ICU stay, of whom 62% (*n* = 25/40) were in the ACP group (Table 3). On Cox univariate analysis, the occurrence of the primary outcome was associated with ACP (*p* = 0.001), age > 65 years (*p* = 0.001), hypertension (*p* = 0.075), and mechanical ventilation (*p* = 0.06). After multivariable adjustment, ACP (HR = 3.35, 95%CI [1.56–7.18], *P* = 0.002) and age >65 years (HR = 2.92, 95% CI [1.50–5.66], *P* = 0.002) remained independently associated with the primary outcome (Table 5). The analysis of survival curves by the Kaplan–Meier curves showed that patients in the ACP group had higher 30-day mortality (*P* = 0.002), especially in the subgroup of non-intubated patients (log rank *P* = 0.04, Figure 2).

**TABLE 5:** Univariate and multivariate Cox regression *analyses* of predictive variables correlated with 30-day mortality in patients with COVID-19-related pneumonia.

Variables	Mortality at 30-days			

	Univariate analysis		Multivariate analysis	
		
	HR (95%CI)	*P*	HR (95%CI)	*P*
Age > 65 years	2.95 [1.59-5.43]	0.001	2.92 [1.50-5.66]	0.002
BMI (for 1 point increase)	0.71 [0.29-1.69]	0.44	-	-
Hypertension	1.75 [0.87-3.65]	0.12	-	-
RV dilatation	0.45 [0.11-1.99]	0.29	-	
ACP	2.85 [1.53-5.31]	0.001	3.35 [1.56-7.18]	0.002
Mechanical ventilation	2.12 [0.91-4.9]	0.06	1.69 [0.72-3.99]	0.22
SOFA CV > 3	1.23 [0.57-2.62]	0.58	-	

ACP, acute cor pulmonale; BMI, body mass index. CV, cardiovascular. HR, hazard ratio. RV, right ventricle; SOFA, sequential organ failure assessment.

## Discussion

The results of our study evaluating the prognosis of ACP in patients with COVID-19-related pneumonia can be summarized as follows: (1) the incidence of ACP was 36%, (2) ACP was present in 38% of non-intubated patients, (3) classical risk factors of ACP in NC-ARDS were not associated with ACP, (4) ACP increased mortality in patients with COVID-19-related pneumonia admitted to ICU, and (5) RV-LSF could be helpful in RV systolic function assessment in this clinical setting.

### Prevalence of acute cor pulmonale in severe COVID-19-related pneumonia

In the era of protective ventilation, the prevalence of ACP in NC-ARDS has been evaluated to be 22% during the first 72 h of mechanical ventilation (1). In our study, ACP occurred in 36% of patients, and 35% (*n* = 32/90) of patients were under mechanical ventilation. This result was close to a study conducted by Cavaleiro et al., who found a high prevalence of ACP (38%, *n* = 44/117, 95%CI 0.29–0.47) in a cohort of CARDS despite protective mechanical ventilation (18). In a smaller sample size study, Bagate et al. found even a higher prevalence (46%, *n* = 31/67) (27).

### Acute cor pulmonale in non-intubated patient and right ventricular dilatation

In our study, 38% (*n* = 20/52) of non-intubated patients with ACP were diagnosed. There are very less data on ACP in non-intubated patients without thrombotic event. Several studies have described RV dilatation and dysfunction (assessed by echocardiography) in COVID-19, but ACP is rarely reported. For example, in a large prospective international survey including 1,272 patients with COVID-19 infection, Dweck et al. reported 33% of RV abnormalities, including 15% of RV dilatation. However, the presence of ACP was not reported (16). In the study by Soulat-Dufour et al., RV dilatation (defined by an RV/LV ratio of >0.6) in non-intubated patients with COVID-19 occurred in 12% of cases (*n* = 47/407). It was independently associated with death or ICU transfer (14). Unfortunately, the presence of septal dyskinesia was not reported. In our cohort, 69% (*n* = 101/146) of patients had RV dilatation, defined by the RV/LV area ratio of >0.6. Previous studies have found an incidence of RV dilatation in patients with COVID-19 admitted to ICU between 30 and 74% (28, 29), depending mainly on the definition used to assess RV dilatation.

### Acute cor pulmonale and pulmonary embolism

In our study, PE was diagnosed on CT scan, before ICU admission, in 8 (5%) patients and was not associated with ACP (2/52 vs. 6/94, *p* = 0.71). Cavaleiro et al. found similar results (8%) in their study and showed that PE was the only factor associated with ACP (18). It is well known that massive PE may induce ACP and require specific medical management (30). However, in the study conducted by Cavaleiro et al., the number of patients with PE in the two groups (2/73 vs. 7/44, *p* = 0.007) is limited, making it challenging to draw any conclusion.

### Classical risk factors for acute cor pulmonale

In NC-ARDS, factors increasing pulmonary vasoconstriction (hypercapnia, hypoxemia, and high driving pressure) under mechanical ventilation are included in the ACP risk score. In NC-ARDS, when two or more risk factors were present, the risk of ACP exceeded 20% (1). In our study, ACP risk score and classical factors were not associated with ACP in patients under mechanical ventilation. Our results were in accordance with a previous study by Cavaleiro et al. Cavaleiro et al. found that the ACP risk score and its components were not associated with ACP (18) in CARDS. They concluded that ACP is likely associated with microangiopathy or thrombosis related to COVID-19 infection (18). To date, no clinical score assesses the risk of ACP in non-intubated patients.

### Right ventricular afterload, pulmonary blood flow, and thrombotic complications

Acute cor pulmonale is the most severe presentation of RV dilatation and dysfunction due to an acute increase in RV afterload. Under normal conditions, RV afterload highly depends on the distribution of blood flow in the lung, the degree of hyperinflation, and the alteration of the pulmonary vasomotor tone (6). Physiological studies have shown that RV is more adapted to rest than stress or exercise. During exercise, the increase in cardiac output increases PVR, consistent with the relationship between mean pulmonary artery pressures and blood flow (the so-called P/Q relationship). This phenomenon promotes RV dilatation (31). In CARDS, the increase in pulmonary blood flow is probably due to pulmonary vessel dilatation and pulmonary neoangiogenesis, leading to perfusion abnormalities toward the areas of diseased lungs, resulting in a worsening ventilation-perfusion mismatch and clinical hypoxemia (12). To cope with COVID-19-related increased pulmonary blood flow, the RV increases its end-diastolic volume by dilatation. Such acute dilatation may lead to ACP. This pathophysiological explanation was emphasized by Caravita et al. in their study using pulmonary artery catheters in patients with CARDS. Caravita et al. showed that PVR was not increased and that a mild increase in pulmonary artery pressure was only explained by an increased cardiac output (11). The decrease in PVR induced by COVID-19 infection (compared to other causes of ARDS) may explain the clinical benefit of administering a selective pulmonary artery vasoconstrictor in patients with CARDS (32, 33). These results emphasize that COVID-19 is a vascular disease that primarily affects pulmonary vessels and induces hypoxemia and RV dysfunction (6, 8). In contrast, COVID-19 infection may also promote pulmonary vasoconstriction. Local pulmonary inflammatory response and vascular endothelial dysfunction induce an immune thrombosis, leading to intravascular clot formation in small and large vessels (34). Thrombotic complications may contribute to causing pulmonary vasoconstriction that worsens RV afterload through a complex interaction between humoral factors, endothelial effects, and hypoxia.

### Right ventricular afterload and left ventricular filling pressures

The increases in RV afterload can be attributed to a downstream factor. Caravita et al. showed that post-capillary pulmonary hypertension and pulmonary artery wedge pressure were higher in CARDS than in NC-ARDS (11). It has been hypothesized that SARS-CoV-2-related myocardial injuries may impair LV diastolic properties (35) and increase left atrial pressures. This increase may be transmitted through the pulmonary circulation to the RV.

### Right ventricular injury and COVID-19

Myocardial injury can result from a direct viral lesion of endothelial and myocardial cells, resulting in endothelial dysfunction, local endotheliitis, and myocarditis (36). Besides, myocardial oxygen supply-demand imbalance in hypoxemia, stress-induced cardiomyopathy, and tissular hypoperfusion may lead to RV dilatation due to myocardial injury (34).

In summary, ACP in COVID-19-related pneumonia is probably the result of an increased RV afterload due to a combination of complex physiopathological factors such as low pulmonary resistance (11), intrapulmonary shunting (12), high cardiac output, increased left atrial pressure (8, 13), and direct myocardial injury.

### Acute cor pulmonale and mortality

Acute cor pulmonale doubles the mortality risk in NC-ARDS, and an RV protective strategy is necessary to avoid hemodynamic failure. In our study, patients with ACP had more frequently developed cardiogenic shock and renal failure, requiring renal replacement therapy. These complications reflect the low cardiac output and venous congestion due to ACP (37).

In our study, the 30-day mortality of ACP was 38% (*n* = 20/52), a similar result to that of Cavaleiro’s study (34%, *n* = 15/44) (18). In our research, ACP was associated with 30-day mortality independent of mechanical ventilation. Our study emphasizes previous studies showing a strong association between RV dilatation or RV dysfunction (ACP combines these 2 RV abnormalities) and poor prognosis in COVID-19 (14, 15).

### Right ventricular-longitudinal shortening fraction at acute cor pulmonale

In our study, only TAD parameters were impaired in the ACP group. A previous study has shown that RV-LSF, a global RV systolic function parameter, was the most accurate parameter for assessing RV systolic function in ACP (26). ACP is characterized by pressure overload, changes in RV chamber geometry, and myocardial dyssynchrony. These factors may influence the accuracy of conventional parameters such as TAPSE or RV-S’ (23). Moreover, the RV-LSF can be a semi-automated and reproducible parameter (26). However, no RV-LSF threshold defines RV dysfunction, even though many studies have found a threshold close to 20% (26, 38, 39). Contrary to other studies (18), our study provided new echocardiographic data in this particular situation, i.e., ACP.

## Limitations

Our study admits several limitations. First, this is a single-center study with a limited sample size. However, it is the first to assess ACP in intubated and non-intubated patients with COVID-19-related pneumonia. Second, we used TTE to assess ACP. The sensitivity of TTE for ACP diagnosis in ARDS under mechanical ventilation is poor compared to transesophageal echocardiography (TEE) (36) and may underestimate the number of ACP. However, in non-intubated patients, TTE is the reference ultrasound method (28) and has good sensitivity and specificity for ACP diagnosis (40). Moreover, TEE may be considered an invasive examination lacking feasibility, especially for hypoxic patients under non-invasive ventilation or high-flow oxygen therapy. Third, we excluded patients under ECMO therapy. Implementing ECMO therapy improves gas exchange and achieves ultra-protective ventilation that may decrease RV afterload and improve ACP. Fourth, in more than 20% of our patients, we could not detect tricuspid regurgitation to evaluate systolic pulmonary artery pressure and arterial-ventricular coupling. Finally, we did not perform pulmonary artery catheters for our patients to accurately measure pulmonary artery pressures and PVR. These data would have been of great interest.

## Conclusion

For critically ill patients with COVID-19-related pneumonia, ACP is frequent (36%) even in non-intubated patients and is associated with increased mortality risk. ACP does not seem to be associated with classical risk factors for these patients. These results need confirmation in further studies with a larger sample size.

## Data availability statement

The raw data supporting the conclusions of this article will be made available by the authors, without undue reservation.

## Ethics statement

The studies involving human participants were reviewed and approved by Amiens University Hospital IRB (Comité de Protection des Personnes Nord-Ouest II CHU-Place V. Pauchet, 80054 AMIENS Cedex 1, CNIL Number: PI2020_843_0026). Written informed consent for participation was not required for this study in accordance with the national legislation and the institutional requirements.

## Author contributions

CB, OA-A, and YM: concept and design. CB, NM, SB, CD, MC, PH, and CV: data acquisition, analysis, and interpretation. CB: drafting of the manuscript. CB, YM, and HD: critical revision of the manuscript for important intellectual content. CB: statistical analysis. YM: supervision. All authors contributed to the article and approved the submitted version.
